# Winter Retreat for Those Inclined to Pulmonary Consumption

**Published:** 1835

**Authors:** 


### XI.—Winter Retreat for those inclined to Pulmonary Consumption.

The gentleman just quoted, has expressed to us the opinion, that the maritime portions of the state of Mississippi, lying below the 31st degree, and between New Orleans and Mobile, constitute one of the best retreats in the United States, for persons who are threatened with pulmonary consumption. They, however, in whom the disease is established, should not leave home for that or any other place in the South, as it has appeared to him, that a southern climate has rather accelerated than retarded the fate of such patients, which is very much the result of our own observation. The soil and climate of the tract of country, to which we have alluded, are described by Dr. C. as sterile, temperate, and comparatively free from humidity.

